# Supportive supervision from a roving nurse mentor in a community health worker programme: a process evaluation in South Africa

**DOI:** 10.1186/s12913-022-07635-w

**Published:** 2022-03-10

**Authors:** Hlologelo Malatji, Frances Griffiths, Jane Goudge

**Affiliations:** 1 Centre for Health Policy, School of Public Health, Faculty of Health Sciences, University of the Witwatersrand, Johannesburg, South Africa; 2 Warwick Medical School, Warwick University, Coventry, UK

**Keywords:** Community health workers, Process evaluation, Intervention, South Africa, Health system integration

## Abstract

**Background:**

Many low and middle- income countries (LMICs) are repositioning community health worker (CHW) programmes to provide a more comprehensive range of promotive and preventive services and referrals to the formal health service. However, insufficient supervision, fragmented programmes, and the low literacy levels of CHWs often result in the under-performance of the programmes. We evaluate the impact of a roving nurse mentor working with CHW teams proving supportive supervision in a semi-rural area of South Africa.

**Methods:**

We conducted a longitudinal process evaluation, using in-depth interviews, focus groups and observations prior to the intervention, during the intervention, and 6 months post-intervention to assess how the effects of the intervention were generated and sustained. Our participants were CHWs, their supervisors, clients and facility staff members and community representatives.

**Results:**

The nurse mentor operated in an environment of resource shortages, conflicts between CHWs and facility staff, and an active CHW labour union. Over 15 months, the mentor was able to (1) support and train CHWs and their supervisors to gain and practice new skills, (2) address their fears of failing and (3) establish operational systems to address inefficiencies in the CHWs’ activities, resulting in improved service provision. Towards the end of the intervention the direct employment of the CHWs by the Department of Health and an increase in their stipend added to their motivation and integration into the local primary care clinic team. However, given the communities’ focus on accessing government housing, rather than better healthcare, and volatile nature of the communities, the nurse mentor was not able to establish a collaboration with local structures.

**Conclusions:**

A roving nurse mentor overseeing several CHW teams within a district healthcare system is a feasible option, particularly in a context where there is a shortage of qualified supervisors to support CHWs activities. A roving nurse mentor can contribute to the knowledge and skills development of the CHWs and enhance the capacity of junior supervisors. However, the long-term sustainability of the effects of intervention is dependent on CHWs’ formal employment by the Department of Health.

**Supplementary Information:**

The online version contains supplementary material available at 10.1186/s12913-022-07635-w.

## Background

Low and middle income countries (LMICs) often have a patchwork of community health worker (CHW) programmes, sometimes led by non-government organizations (NGOs), reaching some communities but not others, focused on specific disease (e.g. HIV/AIDS) or population groups (e.g. child health) [1, 2]. The international calls for universal health care have led some countries to attempt to achieve wider population reach with their CHW programmes [3, 4]. Many LMICs are exploring ways to utilize CHW programmes to respond to wider range of conditions, including non-communicable and infectious diseases [3] and increasing CHWs’ roles in promotive and preventative care [5]. The importance of the CHWs’ role has been given greater prominence and urgency during the COVID-19 pandemic, with CHWs expected to educate the public and to identify possible COVID cases [6–8]. The shift to more comprehensive programmes, in terms of population coverage and health conditions, requires greater supervision to manage, train, mentor and monitor CHWs and to facilitate links with the healthcare system and community structures [9].

South Africa has a long history of CHW programmes starting in the 1940s [10]. Since democracy, CHW programmes, despite considerable fragmentation, have played an important role in extending healthcare services to needy populations [11]. With recent reforms of primary health care services, South Africa is establishing a nationwide CHW programme, covering a more comprehensive range of health conditions than in the past [9, 12, 13]. The CHW programme is known locally as the ward-based outreach team (WBOT) programme [14, 15]). Each WBOT is meant to comprise a team of at least 6 CHWs, a nurse (outreach team supervisor), environmental officer and health promoter. The outreach team supervisor role is to provide field supervision to the CHWs during household visits, ensure they [CHWs] have the resources that they require (e.g. stationary) to fulfil their duties, and help establish team relationship with community structures. Each WBOT, operating within a facility’s catchment area, provides promotive and preventive services to households. CHWs, who were previously working for NGOs contracted by the South African Department of Health (DoH) became members of WBOTs. While the DoH required CHWs to have passed their final school examination in order to be transferred to the new programme, this requirement wasn’t always implemented. As a result, the majority of the CHWs recruited into the new programme had low literacy levels. Similar to other contexts, South Africa has a limited number of health professionals available to be the outreach team leaders who oversee the CHWs, and many CHW teams are functioning without adequate supervision and remain poorly integrated into the healthcare systems [9, 16, 17].

CHWs joining these new teams undertake training on identification of the need for antenatal and post-natal care, monitoring immunization and adherence to long-term medication, screening for malnutrition, TB, gender-based violence, making referrals to health and social services, and following up on patients who need to visit the clinic. The training is delivered in two phases, the first with a written examination (level 1), the second with a practical assessment in the field (level 2) [14]. In Sedibeng District, where this study took place, the CHWs also delivered long term medication each month to elderly or disabled patients.

In this paper we report on a longitudinal qualitative process evaluation, describing the impact of a roving nurse mentor. We describe if and how she built the capacity of the CHW teams and their supervisors, their relationships with both the local health system and community structures, their impact, and whether and how the effect was sustained once the nurse mentor left. We explored the iterative interaction between the context the intervention and the moderating factors that lead to a change. While we will report on the changes in quantitative outcomes elsewhere, it is important to mention here that the intervention led to an increase in the proportion of households who received a visit in the last year from 20 to 30%. Moreover, the CHWs provided care to a greater range of people and performed a greater range of more complex tasks. For further papers on the situational analysis see [9, 18], and tool development see [13].

## Methods

### Study design

We used the Medical Research Council process evaluation framework to guide our study design [19–21]. Initially, we set out the broad parameters of the intervention (see below) which remained constant. We sought to identify and understand the iterative interactions between the intervention, its context, moderating factors and how the intervention changed over time, how these interactions led to impact (or not) and whether this impact was sustained.

To capture change over time, we collected qualitative data in three time periods: 1) prior to the intervention; 2) during the intervention (at three time points); and 3) 6 months post intervention (Table 2). Data collection prior to the intervention formed part of a larger situational analysis conducted from September 2016 to February 2017 in which we studied six CHW teams with different supervision configurations [9, 13]. The intervention organized in three phases of data collection was implemented from August 2017 to November 2018. Our final data collection period was from May to September 2019.

### Intervention design

Our situational analysis demonstrated that CHW teams led by an experienced nurse were well integrated in the healthcare system and received supportive supervision [9]. However, CHW teams led by a junior nurse were poorly integrated into the health system and received insufficient supervision [9]. The latter resulted in relatively poor quality care and low household coverage [18]. We shared these findings with district and provincial stakeholders at an intervention design workshop. Given the national shortage of experienced professional nurses, we agreed our intervention would be an experienced professional nurse (nurse mentor), who would work with two CHW teams which had junior nurses as supervisors, moving between the two teams. If a nurse mentor was, in future, employed by the health services to build the capacity of the CHW teams, intention was the nurse-mentor could then move on to build the capacity of other CHW teams and their supervisors, periodically returning to check on teams that she has already worked with. The nurse mentor was expected to: strengthen the capacity of the CHW team including improving clinical knowledge of the supervisors and the CHWs, their skills in client engagement and providing a role model for supportive supervision; strengthen relationships between the CHW team, their supervisor and clinic staff; and strengthen relationships with community organizations and political structures.

### Nurse mentor characteristics and responsibilities

The appointed nurse mentor had a 4-year nursing degree and 15 years’ experience in nursing of which 6 years was in supervisory roles in other CHW programmes. She assessed the needs of the CHWs and their supervisors. The mentor coached the CHWs and the supervisors on the DoH CHW curriculum and organized for the CHWs to sit the examination and to be assessed in the field. The nurse mentor rotated between the two facilities, allowing the supervisors to take charge of the CHWs in her absence and demonstrate the capability to manage the teams on their own. She initiated activities to facilitate collaboration between the CHWs and facility staff and with community structures (e.g., local political leaders and NGOs). Our process evaluation focused on the four common CHWs activities: household registration, medication delivery, patient follow-up, and community engagement (Table 1).

**Table 1:** CHWs priority activities

Activity	Description
Household registration	Each CHW is expected to register new households to identify individuals or families in need of care. Registration of a household requires the completion of a 9-item questionnaire.
Medication delivery	The CHWs are also responsible for the delivery of medication to elderly patients on a monthly basis. On the 6 months, patients have to return to the clinic for a repeat prescription to be issued.
Patient follow-up	The CHWs are responsible for tracing patients who fail to attend clinic appointments.
Community engagement	The team supervisors are expected to engage with community leaders, local NGOs and services to facilitate collaboration and improve the functioning of the programme.

### Study setting

Our stakeholders advised on the selection of our intervention sites. These were WBOT teams located in rural areas of Sedibeng district, led by junior nurses, with each team expected to provide services to a population of about 6000 with at least 1000 to 1500 households [14]. Sedibeng Health District is relatively affluent by South African standards, yet over 20% of the residents fall below the food poverty line [22]. Outside the urban areas, disadvantaged communities with inadequate shelter, food insecurity and high disease burdens have limited access to services such as clinics, transport, water, and electricity. In the two study sites, located approximately 30 km from the district town, residents’ dwellings consisted mainly of government provided housing (small brick houses) and informal settlements (shacks made of plastic and re-used corrugated iron). The majority of residents in the two sites were unemployed and dependent on government social grants.

### Data collection

Our data collection methods were observations, interviews, and focus groups (Table 2). The first author (HM), a doctoral researcher, undertook data collection prior to the intervention and supervised data collection team throughout the study. The research team trained the data collectors in community orientated health care, qualitative data collection and research ethics. Data collection was in English but where a participant struggled to understand, the team clarified using local languages (Sesotho and IsiZulu). There was no reported refusal to participate in the study. Audio recordings were transcribed verbatim and checked for accuracy by members of the research team.

**Table 2:** Number and type of respondents in each data collection phase

Data collection method	Type of participant	Prior to intervention (2 sites) Sept 2016	During intervention (2 sites) August 2017–November 2018			Six months post- intervention (2 sites) May 2019	Total
			Period 1	Period 2	Period 3		
FGD	CHW	2	–	–	–	2	4
Obs days	CHW (with & without supervisor)	40	7	9	7	24	87
	CHW (with Nurse Mentor & Supervisor)	n/a	16	11	6	n/a	33
In-depth interviews	CHW	–	10	11	4	18	43
	Supervisor	2	5	4	2	2	13
	Nurse Mentor	n/a	–	2	2	n/a	4
	Facility staff members	3	15	5	6	10	39
	Clients	28	11	7	2	–	48
	Community representatives	8	–	–	13	–	21
Reports reviewed	Nurse mentor	–	10	18	8	–	36

The research team fed back initial findings to the CHWs and facility staff participants after the first two rounds of data collection at meetings held at each of the CHW team’s clinic base. These feedback meetings allowed the participants to comment on the study findings, and the research team to refine the next round of data collection [23]. The research team had no prior personal and professional relationship with the study sites and participants.

#### Focus groups

We held 4 focus groups with the CHWs, two prior to the intervention and two in the 6 months post intervention. Each focus group had approximately 10 participants, both men and women, although very few of the CHW were men and all CHWs at the 6 facilities were invited to participate. In the first focus groups, we asked the CHWs about their experiences of the CHW programme, their working conditions, and perceptions of its successes and challenges (see Additional file 4). We also asked the CHWs to complete a short questionnaire about their education and years of experience. Post intervention, we asked about their experiences since the nurse mentor left, and whether their routines had changed. The focus groups lasted between 1 and 2 hrs.

#### Observations

We designed observation templates and refined them through role-plays involving the data collectors (see Additional file 3) [24]. At each site, we observed CHW meetings, household visits and supervision of CHWs, both in the community and in the facility. In the facilities, we observed interactions between patients, facility staff members and members of the CHW team. CHWs worked in pairs for household visits. For observation of these visits, we observed the same pair of CHWs for 3 days in a row, so they became accustomed to the fieldworker being present. The fieldworker asked the household members for permission to observe the CHW.

#### Interviews

We undertook 168 semi-structured interviews with the CHWs and their supervisors and with a purposive sample of health facility staff members, patients and community representatives who were involved with and/or had knowledge of the CHW programme in the district (see Table 2). The interviews lasted 30–60 min each. With CHWs we asked about home visits observed prior to the interview (see Additional file 1). With the supervisor and facility staff members, we asked about their interaction with the CHW team, benefits and challenges of the CHW programme and the intervention. We asked clients and community representatives about the care provided by the CHWs, experiences of care at the facility and relationships between community structures (e.g. NGOs) and CHW programme. We also interviewed the nurse mentor and reviewed the weekly reports she submitted describing the programme’s activities, challenges and achievements.

### Data analysis

We drew together all the data for each team and associated facility. We extracted data from the original transcripts into a word document in chronological order, summarizing data and including verbatim quotations. This process increased our familiarity with the data, reduced the considerable volume of the data and allowed assessment of change over time. The first author (HM) did the extraction and made weekly presentations to the research team, who checked the extracted data against the raw dataset, until the research team was confident that no significant data was being omitted. Once the data extraction was complete, following thematic analysis method [25], we developed a coding system that included CHWs priority activities and emerging themes (such as organizational work systems, staff relationships, CHWs unionisation and healthcare system integration) and coded the extracted data using NVIVO 12 software. The coding was completed by the first author, with weekly discussion with the wider research team. Emerging themes were grouped together using the CHWs priority activities. The coded data was synthesized to understand how the study participants responded to the nurse mentor’s activities meant to improve their performance in household registration, medication delivery, patient follow up and building relations with community structures, and contextual influences and what the outcomes were.

## Findings

### Training and resources

Prior to the intervention, both supervisors had been in their posts for 4 months. They had completed 2-year nursing qualification and 2–3 years post-training work experience but had not worked with CHWs before. One of the supervisors (Team 2) originated from outside the province and sometimes appeared uneasy supervising the CHWs, as the majority of the CHWs were local women and older than her. The supervisor in Team 1 was from the district, assertive and managed a relatively younger group of CHWs (Table 3).

**Table 3:** CHW team members characteristics

Category	Team 1	Team 2
**Supervisor**		
No. of enrolled nurses	1	1
Age (years)	36	31
Mean years as nurse	5	2
Years in programme	0.3	0.3
**CHW**		
No. of CHWs per team	14	20
Mean age in years (range)	42 (23–58)	33 (23–54)
Mean years (range) as CHW	10 (3–9)	6 (5–17)
No. of CHWs who have finished high school education	25%	33%
No. of CHWs who have passed phase 1 training	2	1
No. of CHWs who have passed Phase 2 training	0	0

Prior to the intervention, the CHWs’ length of service ranged between 2 and 4 years. Many of the CHWs had not completed their final school leaving qualification (Table 3). Only a few of the CHWs had completed Level 1 CHW training, and none had completed the Levels 2 or 3 CHW training. Further details on the characteristics of the CHWs are provided elsewhere [18].

During data collection prior to the intervention, the CHW teams were provided with equipment bags (one bag per pair of CHW), containing blood pressure and glucose machines, weight scales, bandages and umbrellas. In one team, the CHWs had not received training on how to use the manual blood pressure machines provided. By the time of the intervention, much of the equipment was faulty. The CHWs shared the remaining working equipment; despite informing the district office, the faulty equipment was not replaced or repaired. The Team 2 CHWs held their work planning meetings in the facility meeting room, however the room was often used by nurses, and then the CHWs had to move outside the facility. CHW commented: “*When they [nurses] like they don’t even tell you that they have a meeting; they just enter the room… you just know that you have to go outside”* (Interview, CHW4, Team 2). Team 1 CHWs, based in a smaller facility, had no room to use, and met their supervisors outside.

Prior to the intervention, according to interviews with CHW team members, CHWs needed to make copies of their various forms that they use during household visits (e.g., household registration and referral forms). However, according to the team supervisors, the photocopy machines were often broken or out of ink. Staff had to contribute their own funds to purchase ink, which many of the CHWs could not afford, and so didn’t make copies. The supervisors had to travel to the sub-district office (30kms away) to make copies. (During the intervention, the nurse mentor occasionally provided copies of stationary so the CHWs were able to undertake their work). CHWs had to use their own funds to purchase a notebook and pen to record daily activities; many used loose pieces of papers to record the details of visits. Due to space constraints within the two facilities, the CHWs kept completed client forms at home. This practice had a negative impact on the CHWs’ work, as the forms were rarely brought back to the clinic and were not used to reporting CHW activities.

### Conditions of employment and unionisation

At the beginning of the study, the CHWs were a contracted labour force managed by a private administrative payroll company. They received a stipend of R2 500 (143 USD) per month. The facility staff members expressed dismissive attitudes towards the CHWs. A CHW commented: “*The facility manager tells us that we are not part of the clinic [because they were contracted to the payroll company] so there’s nothing she can do for us’* (Interview, CHW8, Team 1). The CHWs felt belittled: “*The peer educators, HIV/AIDS counsellor we all go together to sign the same contract, but they are treated as if they are more educated than us, they call us street maids”* (CHW-FGD, Team 2).

A task team was established by the CHWs to demand improved conditions of employment. The task team consisted of CHWs, lay counsellors and health promoters from the district. Only a few CHWs from our study sites participated in the task team meetings, as they were held in the district town and transport was expensive. A greater number of the CHWs participated in protests, which were often 1 day ‘stay-aways’; one militant CHW threatened to report CHWs to the task team if they went to work. Clinic staff often asked the supervisors and CHWs to do facility-based work when they should be in the community; the CHWs were told by the task team to stop activities in the facilities, including those activities that were part of the CHW programme (e.g., assisting nurses to retrieve CHW patient files and practicing taking blood pressure measurements in the vital signs room). They were also told not to work when the supervisors were not present, or if it was raining.

Towards the end of the intervention, the CHWs were formally employed by the Provincial Department of Health (PDoH) in June 2018. Their monthly stipend was increased to the minimum wage of R3 500 (200 USD). The increment encouraged the CHWs: “*It has motivated me to work harder than before”* (Interview, CHW, Team 2). Some CHWs used the increment to invest in the education of their children: “*I am now able to save for my child secondary school education. I have been saving R1 000 every month, so when she passes matric I am able to pay for her college fees*” (Interview, CHW5, Team 2).

The following sections focus on the findings from the four focal areas of the nurse mentor intervention – household registration, medication delivery, patient follow-up, and community engagement.

#### Household registration

##### Prior to intervention

Prior to the intervention, the number of households being registered was low. Moreover, the CHWs often asked less than half of the nine household registration questions, partly because they did not understand the questions as they are written in English, or the purpose of the questions.

##### During the intervention

The mentor gave training sessions, facilitated role plays where the supervisors and CHWs could practice engaging with household members, and accompanied them on household visits, supporting them as they practiced their new skills. The mentor supported and supervised the two supervisors, demonstrating how to provide supportive supervision during household visits with the CHWs. In early training sessions and household visits, one supervisor was reluctant to participate in activities. The nurse mentor commented “*At first [the supervisors] were struggling because they did not know the content themselves especially the supervisor from Team 2. She was frustrated.”* (Interview, Nurse mentor).

Several of the CHWs were showing resistance to receiving instruction from the nurse mentor and were obstructive or often absent. However, the CHWs came to appreciate the assistance they received from the mentor: “*At first I was scared of the nurse mentor but now I enjoy learning new things from her”* (Interview, CHW3, Team 2). The mentor took time to unpack complex topics: “*She was giving a lesson about a particular health condition. I could not understand her, so I approached her. She sat me down and went over the lesson until I understood”* (Interview, CHW6, Team 1). This patience relieved some of the CHWs anxieties. However, when the mentor felt the CHWs had not paid attention or applied themselves sufficiently, she would get irritated. “*The nurse mentor sometimes shouts at me in front of patients when I make mistakes. She doesn’t keep quiet and let me finish what I am doing and correct me later, she shouts at you right there”* (Interview, CHW8, Team 1). In protest, some withdrew from the training, and reported to the task team that the nurse mentor is forcing them to participate in-service training against their will. This resulted in a tension, with the mentor and supervisors being threatened by task team members when attending district meetings, particularly when they raised issues relating to CHWs poor performance. The mentor adopted a gentler approach, which helped soften the stance of the CHWs; overtime the CHWs came to realise the mentor didn’t want to intimidate them, rather to ensure they worked to improve their performance “*the nurse mentor is the type of person who just want to see progress in your work.*” (Interview, CHW2, Team 1).

##### End of intervention

The majority of the CHW passed both the Level 1 and 2 training as a result of the nurse mentor’s coaching; only 1 in Team 1 and 3 in Team 2 failed due to their low level of literacy. These individuals were moved to a home-based care programme. The training, and passing their examinations, boosted the CHWs’ morale: “*When I get to a household, I don’t feel ashamed anymore, I enter with confidence because I know my work”* (Interview, CHW7, Team 1). Patients expressed their appreciation: “*The patient told the mentor and supervisor that she is happy with the CHWs. Before the intervention, the CHW’s visit was brief. The visits now take longer and the [CHWs] monitor her BP and sugar level”* (Interview, Client 3, Team 1). The supervisor, who appeared passive and defensive early on, grew in confidence and began to take initiative: “*She is very active and engaging when supervising the CHWs. She informs them if they have not given a patient appropriate health information during their visits”* (Nurse mentor; interview).

##### 6 months post-intervention

In the post-intervention period, the supervisors continued to accompany the CHWs on household visits: “*She assists us; recently there was a problem with one lady who had not brought her children to the clinic for vaccination. The supervisor went to the household with the CHWs to speak to her.”* The woman, subsequently, took the children to the facility (Interview, CHW5, Team 2). The supervisor for Team 2 adopted a sensitive approach when supervising the CHW: “*She prefers to keep quiet while in the households even if you make mistakes. It is only when we meet at the clinic that she will identify your errors and advise you how to fix them”* (Interview, CHW2, Team 2). The CHWs appreciated this approach.

The supervisors continued to provide training to the CHWs every Friday: *“Last week Friday, she trained us on pregnancy screening, STIs, HIV and heart attack”* (FGD, CHW, Team 1). When asked, CHWs prepared informative lessons on a given topic to share with their colleagues.

#### Chronic medication delivery

##### Prior to intervention

The medication delivery process was as follows: a CHW collects a patient’s clinic card from the patient’s home and brings it to the clerk at the facility who retrieve the patient file. The file goes to a professional nurse who confirms the patient’s repeat prescription. The pharmacy assistant prepares the medication for delivery. The CHW takes the medication to the patient at their home and measures and records the patient’s BP or blood glucose on the card. In the pre-intervention period, some patients were not receiving their medications on their scheduled dates and would come into the clinic to complain, or the CHWs, realizing they had missed a patient’s date, would hurriedly request for the medication from the facility staff. Both outcomes led to tension between the CHWs and facility staff: “*Sometimes they will expect you to pack the medication the same day they are expected to deliver..,you have to stop what you are doing and attend to them”* (Interview, Pharmacy Assistant, Team 1).

##### During intervention

The mentor engaged with all staff involved to understand the challenges with the medication delivery system. She trained the CHWs to record the details of patients (name, address, type of medication, expected delivery date and CHW return date) in a book. She enlisted the support of the clinic staff (nurses, pharmacy assistants and clerks) for the new system she put in place in the facilities. The staff members agreed to their different roles.

Despite the new system, some CHWs still collected the appointment cards late. One clerk insisted that the CHWs queue like an ordinary patient to get a patient file. The mentor negotiated with the manager in Facility 2 for two CHWs to assist in retrieving files to minimize the delay, and for the professional nurses to issue the medication. This helped ease the frustrations of the clerks and pharmacy assistant. In Facility 1, there was insufficient space for CHWs to assist in the filing room, but the facility manager stepped in to resolve conflicts where possible.

In the two facilities, the nurse mentor spent time negotiating with the facility managers for the inclusion of the CHWs in facility meetings, as this was a potential forum to discuss and resolve issues surrounding medication delivery. However, the managers refused, as the CHWs were contracted to an external pay roll company and did not see the CHWs as their responsibility. In Facility 2, the nurse mentor did negotiate for the CHWs to help take the patients BP measurements in the clinic, so they could practice their skills and gain confidence. However, the facility manager appeared distrustful of the CHWs and her attitude allowed her junior staff to dismiss the CHWs efforts, rather than train the CHWs: “*Yuuu that was a disaster. They did not know what they were doing, only few of the CHWs were trying. I had to stop them from coming as they overcrowded the room”* (Interview, Enrolled Nurse Assistant, Facility 2). In Facility 1, the facility manager was keen to have the CHWs in the triage room to help take patients BP measurement, however, the room was too small for this.

##### End of the intervention

Recording the patients’ delivery dates in their books helped the CHWs ensure they delivered the medication on time, and the facility staff noted the improvement: “*They have improved because now they take the dates of all the patients. Every week they take out the files for the whole week and give them to their team leader who then takes them to the pharmacy room. The pharmacy assistant packs the medications for the CHWs for the whole week”* (Interview, Enrolled Nurse Assistant, Team 2). The lack of equipment continued to hinder the CHWs’ ability to do their job: “*The blood pressure and glucose machine broke. Now, I just deliver [medication] without doing anything to the patient”* (Interview, CHW6, Team 2). Some patients demanded they monitor their vital signs; the CHWs reported being embarrassed that they could not.

Towards the end of the intervention, when the CHWs were formally employed by the DoH, the facility managers became responsible for the CHW teams and the CHWs started to participate in clinic meetings. A nurse commented: “*It makes a huge difference because we get to learn what it is that they are doing out there, the challenges they face. Once everybody is involved in the meetings, it means we can all take ownership of our work”* (Interview, Professional nurse, Team 2). The professional nurses acknowledged the change in CHW’s performance and the CHWs felt supported by them: “*We work well with facility staff. Whenever we want patients’ files they assist us on time. If I am not feeling well, I am able to speak to the nurses and get medication without having to join the queue like a patient would do”* (Interview, CHW9, Team 2).

##### 6 months post intervention

In the post-intervention period, the CHWs continued to collect patient appointment cards mostly on time, and the supervisor, professional nurses and pharmacy assistants cooperated with the CHWs. However, some of the CHWs occasionally forgot to collect the patient appointment cards and were penalized by their supervisors: “*You have to join the queue to get the patient medication…she does not take stories*” (Interview, CHW2, Team 2).

#### Patient follow- up

##### Prior to intervention

The CHWs’ difficulties in locating and persuading patients to return to the clinic affected their relationship with facility staff members who believed the CHWs did not put sufficient effort into tracing patients. However, some nurses were not mindful about ensuring patient confidentiality, particularly for HIV treatment, and as a result, patients sought care in clinics outside their own community. Knowing nurses would insist that they return to their local clinic, patients often give an incorrect address, hampering the CHWs efforts to locate them. Others, once located, refused to return to clinic: “*I do not see the reason to go to the clinic, nurse A does not treat people well, she walks around publicly displaying our medication to staff members and patients”* (Interview, Client 3, Team 1).

##### During intervention

The nurse mentor arranged for the clinic clerk to provide a list of names and residential addresses of all patients who needed to be followed up each week. It was agreed that CHWs should visit each address at least three times on separate days before marking the patient as untraceable and would give feedback on their progress a week later. As a result, it became clear what was expected of the CHWs, and they could mark a patient as untraceable, rather than continued to be blamed for the non-appearance of the patient. The nurse mentor accompanied CHWs to visit patients who were refusing to return to the clinic: “*Even if she [nurse mentor] is with another CHW, if I encounter a problem, I am able to contact her for assistance*” (Interview, CHW8, Team 1). The household visit by the mentor often reassured the patient and helped resolve any impasse between the patient and clinic.

##### End of the intervention

The CHWs efforts at tracing patients improved, and the data clerks provided the CHWs with regular support. As the CHWs were acknowledged as contributing members of staff, the relationship with staff improved. One facility manager commented: “*They are very helpful. In our facility we have many patients who have defaulted on their medications. The CHWs help us locate these patients*” (Interview, Facility Manager, Team 1).

##### 6 months post intervention

Post intervention, the facilities reported slight drop in the number of patients returning to care. One facility had fewer antenatal and postnatal care patients and the manager attributed this to CHWs inability to successfully locate cases and provide feedback. However, the supervisors were able to provide support with challenging cases, as the mentor had done: “*There was a teenage boy who was on ART medication and refusing to take the medication. The supervisor accompanied me to the boy’s home. She persuaded the patient to go the facility”* (Interview, CHW9, Team 1). The mentor intervention had improved the supervisor’s skills at engaging with patients.

#### Engagement with community structures

##### Prior to intervention

Prior to the intervention, the CHW programme had little engagement with community leaders and representatives (e.g., ward councilors). Most community leaders knew little about the programme, and there was no collaboration with local NGOs (e.g. those providing food) who might have identified households in need of health care.

##### During intervention

The nurse mentor repeatedly talked to ward councilors about setting up meetings with the community but only managed to hold one meeting. These local political leaders appeared disinterested in supporting the mentor in creating a link between the CHW programme and community; several meetings were agreed upon but later cancelled or postponed. Community meetings called by the ward councilors were to discuss the lack of housing and sanitation, rather than health care. Some of these meetings turned violent as the residents wanted the councilors to speed up the process of providing housing and other services of immediate concern. The service provided by the CHWs was not a priority.

Following nurses’ complaints that traditional healers give pregnant women traditional herbs to manage their pregnancy, the mentor organized a meeting with traditional healers. A traditional healer participating in the meeting narrated, “*They were complaining that we [traditional healers] give pregnant women ‘isihlambezo’ (traditional herb) and that the babies are born disabled. It is not the herb that causes the disability but other things such as drinking and smoking during pregnancy* (Interview, Traditional healer 2, Site 1). The traditional healers were not ready to work with the health facility: “*They [nurses] undermine us”* (Interview, Traditional healer 3, Site 1); a long-standing impasse between the health facility and the traditional healers frustrated the mentor’s efforts to establish a collaboration.

The nurse mentor encountered challenges with uncooperative NGO representatives: “*We went to a meeting of NGOs, there were only few NGO staff present, the meeting had to be postponed because there was no point in continuing with only the few of us present. Till today we have not received a new date for a meeting* (Observations, CHWs, Site 1).

The CHWs had a positive relationship with individual community members, who often informed CHW about individuals in need of their services. A CHW shared: “*When you are walking in the community, community members will tell you to go and attend a sick person in a particular household”* (Interview, CHW11, Team 2). Individuals often approached CHWs informally to seek health-related information: “*Last month I came across [in the street] two gentlemen of which one told us that his body was itching and could not sleep at night.… We wrote him a referral letter to go to the clinic*” (Interview, CHW3, Team 2).

##### End of intervention and 6 months post-intervention

The nurse mentor was not able to establish collaborative relationships within the community leaders and structures.

## Discussion

In our study, we examined the role of a roving nurse mentor in building the capacity of two CHW teams led by junior nurses in two primary health facilities in a semi-rural area of South Africa. Initially, with many of the CHWs not having finished their schooling, the mentor’s involvement evoked fear in some CHWs, resulting in them being obstructive, or not fully participating in capacity-building activities. The mentor had to strike a balance between pushing the CHWs to try to learn and adopting a gentler approach that didn’t alienate them. Over time the nurse mentor was able to get this balance right and the CHWs and their supervisors improved their skills and confidence. Other studies in LMIC settings have similarly found many CHWs may not have finished their school education and stressed the importance of providing a supportive environment to help them overcome their fears of failing again, so enabling them to reach their potential [26, 27].

The nurse mentor negotiated with the staff to establish three new operational systems to assist the CHWs: the book for recording the patients’ medication delivery dates; working in the vital signs room to practice taking BP measurements; and the use of a list and three visits only to patients who needed to be traced. These systems led to an improvement in CHW performance, although the CHWs were still hampered by the lack of equipment and limited space and the dismissive attitude of junior facility staff. The CHWs needed a dedicated senior person to work out what systems were required, negotiate with facility staff to establish them, and to navigate problems when they arose. The change to being employed by the DoH meant the facility managers took greater responsibility for the CHW team and were able to build on the improvements established by the nurse mentor and the programme became more integrated into the facility. It is unlikely that facility managers, with their workload, would have been able to bring the CHWs’ skills and confidence up to the necessary level without the nurse mentor.

Difficult relationships between CHWs and other healthcare staff members have been documented in other studies. In a study of CHWs and professional nurses’ relationships in South Africa [28], the CHWs reported finding it uncomfortable working with professional nurses, as the nurses often failed to recognize CHWs as members of the health team. Other studies have found some clinicians tend to undermine and marginalize the CHWs role [28, 29]. Systematic review evidence suggests that health workers’ negative attitudes towards CHWs affect their performance [30]. A study in Malawi found clinicians who were reluctant to give drugs to health surveillance assistants hindered the health surveillance assistants role and performance in the community [31]. Similarly, Payne et al argued the stalemate between clinicians and CHWs is largely due to differences in training (curative and non-curative) [32]. In our study, we found that a senior nurse, who serves as a point of authority within the CHW teams, and champions the role of CHWs, can be a critical resource in establishing operational systems, and addressing conflicts between CHWs and clinic staff. Similarly, a study in rural north west South Africa, found team leaders were the source of support for CHWs as the facility managers often struggled to provide supervision support due to unmanageable workloads in the facilities [33].

World Health Organization (WHO) recommendations and evidence from several countries suggests that community members tend to utilize services if the health programme is embedded in the community structures [34–37]. For example, in Rwanda village leaders and community security officers had a crucial role in ensuring mothers and pregnant women were aware of the maternal and child services available to them at health facility and community level [38]. In Malawi, volunteers, who belonged to a wide range of community-based committees, supported the health surveillance assistants in completion of their daily tasks and made effort to inform them of problems that required their attention in the community [36]. The communities where we undertook our study had community forums but at the time of the study these focused on the community’s pressing concerns of lack of housing rather than the delivery of healthcare services.

The WHO Global Strategy on Human Resources for Health emphasizes the need to align CHW initiatives and programmes to broader national health workforce policies [39] if CHWs effectiveness is to be realized [34]. Many CHW labour groupings have focused on securing permanent employment, decent wages and recognition of CHWs as contributing members of healthcare system [40–42]. Our findings show that paying CHWs and integrating them into the healthcare system was important for improved and sustained CHWs motivation and performance. The WHO guidelines do not adequately acknowledge the chronic shortage of health workers in LMICs to oversee CHW programmes [9, 30, 35]. Our study provides evidence for the success of a CHW supervision configuration that is potentially suitable for contexts with a health worker shortage. A roving, experienced nurse mentor can be responsible for several CHW programmes in a district healthcare system, contribute to the knowledge and skills development of the CHWs, and enhance the capacity of junior supervisors. However, the long-term success of this approach is dependent on fair remuneration and the integration that results from formal employment of CHWs.

This study contributes to understanding how to address the challenges inherent in many CHW programmes of insufficient supervision, poor health systems integration and poor relations with local communities. During our study, the CHW team and facility staff may have changed their normal routines and behaviors during observations. However, our extensive data collection meant the research team became a familiar presence over the course of the study. The intervention study was undertaken with only two CHW teams so we do not know if the intervention would work if the mentor took on more teams.

## Conclusion

In a resource constrained setting like South Africa, where there is a shortage of health workers to oversee the implementation of CHW programmes, a roving nurse mentor working with more than one CHW team can successfully improve CHWs skills and confidence, the supervisory ability of the CHW team leader, set up appropriate organizational systems, improve the working relationships between the CHW team and health facility staff. However, the long term success of CHW programmes is dependent on formal employment and better integration of CHWs into healthcare systems.

## Supplementary Information

**Additional file 1.** Interview guide for community health workers.

**Additional file 2.** Interview guide for outreach team leaders.

**Additional file 3.** Observation template for community health worker activities.

**Additional file 4.** Focus group discussion guide for community health workers.

## Data Availability

Dataset is available on a reasonable request. Given the difficult of anonymizing qualitative data, we will work with any researchers wishing to further analyze the data. The request to access the dataset can be directed to Professor Jane Goudge, email: Jane.Goudge@gmail.com

## Abbreviations

CHW: Community Health Worker
LMIC: Low and middle-income country
DoH: Department of Health
PDoH: Provincial Department of Health
WBOT: Ward-based Outreach Team
WHO: World Health Organization
