# Supplementary material for: Supportive supervision from a roving nurse mentor in a community health worker programme: a process evaluation in South Africa
Source: BMC Health Serv Res. 2022 Mar 10;22:323. doi: 10.1186/s12913-022-07635-w (PMC8908295; doi:10.1186/s12913-022-07635-w)
Supplement: Supplementary file 1 — Additional file 1. Interview guide for community health workers. [file 12913_2022_7635_MOESM1_ESM.docx]

**Additional file 1: Interview guides for community health workers**

**Thank you for letting me observe your work over the past few days. I just have a few additional questions for you**

**Are there additional things you do that I have not observed?**

**What challenges do you face in doing your work?**

**OUTREACH TEAM LEADER (OTL)**

**Does the OTL understand your work or challenges you face? Do you discuss your work with her? When?**

Probe:

- ask your feedback at the end of day
- organize daily or weekly debriefing
- during in-service training
- in casual conversations (when and where and how often?)
- does she check your notebooks / examine your stats / read patient files

**How does the OTL assist you in your every-day work?** Please give examples

Probe:

- assist with home visits (& how often),
- compile stats (& how often),
- ensure availability of medication and other supplies,
- plan with CHWs regarding how they arrange a day/week’s schedule,
- resolve issues or conflicts with clinic staff,
- attend patients with complex issues
- teach you how to provide better care for patients
- correct you when she sees you doing something wrong

**NURSE MENTOR**

**Does the nurse mentor understand your work or challenges you face? Do you discuss your work with her? When?**

Probe:

- ask your feedback at the end of day
- organize daily or weekly debriefing
- during in-service training
- in casual conversations (when and where and how often?)
- does she check your notebooks / examine your stats / read patient files

**How does the nurse mentor assist you in your every-day work?** Please give examples

Probe:

- assist with home visits (& how often),
- compile stats (& how often),
- ensure availability of medication and other supplies,
- plan with CHWs regarding how they arrange a day/week’s schedule,
- resolve issues or conflicts with clinic staff,
- attend patients with complex issues
- teach you how to provide better care for patients
- correct you when she sees you doing something wrong

**Are there any activities for which you would like more support? Please describe**

**How do you referral patients when they come to the clinic? How well does the referral work?**

Probe:

- make plans when the referral is made
- report to anyone, such as OTL, nurse mentor, staff nurse in the clinic
- make the patient file ready beforehand
- are you required to make follow-up visit? If so, do you do that, and after how long?
- give an example how it goes, and at which point problems might arise and whether you are able to get help…

**How is the relationship between CHW and the facility?**

Probe:

- how is the attitude of the clinic staff towards the CHWs in general?
- do CHWs get a place inside the clinic to meet and do paper work without being chased away
- do CHWs help in the facility, if so, what kind of work and how often,
- If CHWs help clinic work, does the request come from the facility manager, any facility staff, or through the OTL?
- In what circumstances do CHWs need collaboration from facility staff? Please describe.
- Do you always receive help from the clinic when you need? Please give examples.
- Does any facility staff supervise CHWs’ work? Please give examples.

**How is the relationship between CHWs and the community?**

Probe:

- Do you do health campaigns? When was the last time you participated in one?
- Do community members ask for CHW’s help when you are traveling in the community?
- Do you know the councillor?
- Do you know people in the clinic committee?
- Do you receive complaints about WBOTs? What are the complaints about?

**Is there anything else you can tell me that will help me get a full picture of your work at the moment?**

**Thank you for taking part in the research.**
