# Supplementary material for: Supportive supervision from a roving nurse mentor in a community health worker programme: a process evaluation in South Africa
Source: BMC Health Serv Res. 2022 Mar 10;22:323. doi: 10.1186/s12913-022-07635-w (PMC8908295; doi:10.1186/s12913-022-07635-w)
Supplement: Supplementary file 2 — Additional file 2. Interview guide for outreach team leaders. [file 12913_2022_7635_MOESM2_ESM.docx]

**Additional file 2: Interview guide for outreach team leader**

**Introduction**

Thank you for agreeing to be interviewed. Are you ready to start with the interview?

**I would like to ask you some questions about yourself:**

- How many years have you worked as a nurse?
- How many years have you worked at this facility?
- What is your professional title?
- What is your current role?
- How many years at current position?
- What previous work experience do you have as a nurse
- Were you born in this district? / where are you from ?

**I would like to know details about the many areas of your work. Please can you tell me how you undertake each area of work? We are interested in how things are now.**

- Planning what the CHW do
- Supervision of CHW clinical work
- Training CHWs
- Putting together statistics of CHW activity

**What role do you have in relation to?**

- Referrals the CHW make: how are you involved with these?
- Contact tracing: how are you involved with this?
- Medication delivery: how are you involved with this?
- Records of CHW activity (e.g. registration forms, referral forms, notebooks): what do you do with these?

**How do you undertake?**

- Community engagement activities
- Health campaigns
- Working with other organisations in the community
- Building relationships with the other facility staff

**What is your role in securing?**

- Supplies for the CHW team
- Facilities/resources for CHW including space, transport, airtime
- Safety of CHWs

**I would now like to ask you about working with the nurse mentor**

Please describe for me the activities you have done with the nurse mentor in the last week

- Please describe tasks where her input / training /support has been helpful?
- Can you describe some tasks/ activities where you wish you could have had more support?

**I would now like to ask you about team relationships which are not always easy.**

- Having a new leader can be difficult. In your view, how are relationships in the WBOT? Prompts: What is working well; what is not working well?
- Relationships between clinics and WBOT are also not always easy. Having a new member in a clinic / team can be difficult.
- In your view, how are relationships between clinic and WBOT? Prompts: What is working well; what is not working well?

**Thank you for taking part in the research.**
