# Supplementary material for: Supportive supervision from a roving nurse mentor in a community health worker programme: a process evaluation in South Africa
Source: BMC Health Serv Res. 2022 Mar 10;22:323. doi: 10.1186/s12913-022-07635-w (PMC8908295; doi:10.1186/s12913-022-07635-w)
Supplement: Supplementary file 3 — Additional file 3. Observation template for community health worker activities. [file 12913_2022_7635_MOESM3_ESM.docx]

**Additional file 3: community health worker household observation template**

|  | | | | | |
| --- | --- | --- | --- | --- | --- |
| **Observer Name: ___________________________________________________** | | | | | |
| **Observation date:___________________________** | | | **Site: ___________** | | |
| **Unique Observation ID: _____________________________________________** | | | | | |
|  | | | | | |
|  | | | | | |
| **Details of CHWs being observed:** | | | | | |
| **CHW 1: Unique ID : _________________________ (not CHW real name)** | | | | | |
| **CHW 2: Unique ID: __________________________ (not CHW real name)** | | | | | |
|  | | | | | |
| **Observation guidelines:**   - The researcher will record plans made by CHW before household visits: what actually happens during the visit, and what the CHW do when they come back from the field. - CHW work related discussions on the way to the households will also be recorded. As well as the physical surrounding of the community and type of patient dwelling. No pictures or videos will be taken. - Resources in CHWs position such as blood pressure machine, bandages, scale, pen, notebooks, flip charts etc. will be noted down. - Type of activities (or roles) that CHWs assume during household visits will also be an area of interest. - CHWs relationships within patients and other health workers at the facility will also be an area of focus. - OTL supervision of the CHW will also be recorded in the notes. - Changes to planned activities and why changes were made will also be documented. - Other successes and challenges confronting the CHWs and OTL during household visits | | | | | |
| **Activity 1: (as soon as CHW arrive at the facility)**  Please describe activity type (e.g. feedback, supervision session): _____________________________ | | | | | |
| **Start Time:** | **Location:** | | | | |
| **CHWs:** |  | | | | |
| Notes | | | | | |
|  | | **End Time:** | | | |
| **Activity 2:**  Please describe activity type (e.g. walking to the first household): _____________________________ | | | | | |
| **Start Time:** | | | | **Location:** | |
| **CHWs:** | | | |  | |
| Notes | | | | | |
|  | | | | | **End Time:** |
| **Activity 3:**  Please describe activity type (e.g. medication delivery): ____________________________________ | | | | | |
| **Start Time:** | | | | **Location:** | |
| **CHWs:** | | | |  | |
| Notes | | | | | |
|  | | | | | **End Time:** |
