# Supplementary material for: Supportive supervision from a roving nurse mentor in a community health worker programme: a process evaluation in South Africa
Source: BMC Health Serv Res. 2022 Mar 10;22:323. doi: 10.1186/s12913-022-07635-w (PMC8908295; doi:10.1186/s12913-022-07635-w)
Supplement: Supplementary file 4 — Additional file 4. Focus group discussion guide for community health workers. [file 12913_2022_7635_MOESM4_ESM.docx]

**Additional file 4: focus group discussion guide for community health workers**

**I am now going to ask you about your work with WBOT. Please all help to answer the questions. I would like you to tell me about what you do day to day.**

- Please tell me about how a typical day at work starts, what do you do?
- How do you decide which households you’ll visit on a particular day? (do you do this with your colleague(s) who pairs up with you?)
- How do you plan your work at the beginning of each day?
- How do you keep track of the households where you need to follow up on a patient?

**Please can you describe for us the data reporting system here?**

- What information do you record when you are out in the community?
- What do you do with this information, and when?

**Thank you. Now please tell me about your pack.**

- What is in it?
- When do you use the contents? What for?

**What other resources are available for you - e.g. airtime, a meeting place, a place to store your files? Are these sufficient? If not, please explain?**

**What are the challenges that you face (e.g. stipend, appreciation for work done, statistics compilation, work hours, work related conflicts etc.)?**

**What support do you receive from your colleagues, team leader, the clinic staff, from the community? Is the help sufficient? Please explain?**

**What achievements in your work are you proud of?**

**What happens when a team member is struggling or can’t visit all of their households, for example because of sickness?**

**How do you relate to the community?**

**Thank you for taking part in the research.**
